# Uncovering hidden and complex relations of pandemic dynamics using an AI driven system

**DOI:** 10.1038/s41598-024-65845-0

**Published:** 2024-07-04

**Authors:** Umit Demirbaga, Navneet Kaur, Gagangeet Singh Aujla

**Affiliations:** 1 Department of Medicine, University of Cambridge, Cambridge, CB2 0QQ UK; 2 European Bioinformatics Institute (EMBL-EBI), Cambridge, CB10 1SD UK; 3 Department of Computer Engineering, Bartin University, 74110 Bartin, Turkey; 4 Department of Biochemistry,Guru Gobind Singh Medical College and Hospital, Baba Farid University of Health Sciences, Faridkot, Punjab 151203 India; 5 Department of Computer Science, Durham University, Durham, DH1 3LE UK

**Keywords:** Computer science, Scientific data

## Abstract

The COVID-19 pandemic continues to challenge healthcare systems globally, necessitating advanced tools for clinical decision support. Amidst the complexity of COVID-19 symptomatology and disease severity prediction, there is a critical need for robust decision support systems to aid healthcare professionals in timely and informed decision-making. In response to this pressing demand, we introduce BayesCovid, a novel decision support system integrating Bayesian network models and deep learning techniques. BayesCovid automates data preprocessing and leverages advanced computational methods to unravel intricate patterns in COVID-19 symptom dynamics. By combining Bayesian networks and Bayesian deep learning models, BayesCovid offers a comprehensive solution for uncovering hidden relationships between symptoms and predicting disease severity. Experimental validation demonstrates BayesCovid ’s high prediction accuracy (83.52–98.97%). Our work represents a significant stride in addressing the urgent need for clinical decision support systems tailored to the complexities of managing COVID-19 cases. By providing healthcare professionals with actionable insights derived from sophisticated computational analysis, BayesCovid aims to enhance clinical decision-making, optimise resource allocation, and improve patient outcomes in the ongoing battle against the COVID-19 pandemic.

## Introduction

Recently, the globe has faced a pandemic called COVID-19, stemming from an acute respiratory syndrome first identified in late December 2019 in Wuhan. COVID-19 is transmitted between people by respiratory droplets and aerosols^1^, as well as if someone touches their eyes, nose or mouth after touching surfaces where a sick person has recently coughed or sneezed as this virus can survive for several days on a suitable surface at room temperature^2^. To diagnose COVID-19, healthcare staff collects a sample from the nose (nasopharyngeal swab), throat (throat swab), or saliva from patients with distinct symptoms. Similarly, people can diagnose themselves if they are positive for COVID-19 at home through the test kits authorized by the Food and Drug Administration (FDA) (https://www.fda.gov/).

Artificial intelligence (AI) technologies (i.e., machine learning (ML) and data mining) and probabilistic models are crucial in predicting and analysing COVID-19 trends in many areas. Various ML algorithms and statistical methods are used in future predictions from COVID-19 big datasets, and they are actively used in academia and industry to reveal unknown facts. Here are some areas where AI technologies and tools are implemented in the COVID-19 crisis, such as understanding the virus and revealing its hidden aspects, detecting and diagnosing the virus, assisting doctors in planning the treatment of patients with COVID-19, predicting the spread and evolution of the virus, and following the COVID-19 infection and developing early warning systems to this end^3^. One of healthcare organisations’ biggest challenges is accurately diagnosing patients and implementing effective treatments at affordable costs^4^. The ability to analyse the millions of patients’ health records empowered by AI and statistical methods can solve fundamental and critical problems regarding patient treatment and hospital costs.

King’s College London-led researchers have discovered, through analysis of COVID-19 data, that there are six different “categories” of COVID-19, each of which can be identified by a specific cluster of symptoms^5^. This study aimed to help healthcare staff predict the patients at risk and who should be hospitalised. Findings from this study show that in addition to persistent cough, fever, and loss of smell, which are shown as the three main symptoms of Covid-19, it can be seen in various symptoms such as headaches, shortness of breath, runny nose, sore throat, diarrhea, hemoptysis, etc. Moreover, in this study, a ML algorithm was applied to the dataset of approximately 1600 patients with COVID-19 from the USA to find out which symptoms tended to appear together. The research identified six specific ’types’ of COVID-19 or particular groupings of symptoms that manifest at distinctive stages throughout the illness. After that, a second independent dataset of 1,000 COVID-19 patients from the UK, US, and Sweden who had recorded their symptoms was used to evaluate the algorithm. Table 1 shows six clusters identified by the researchers. The most important question, however, here is: *“How can we turn this information into meaningful insight?”*. Uncovering the relationship between these symptoms in all COVID-19 patients worldwide may open the door to new research. However, the large size and highly heterogeneous nature of healthcare data, often missing, render it relatively challenging to reveal relationships between features. Therefore, manual time and human expertise are required to preprocess the data and uncover hidden relationships.

**Table 1:** COVID-19 severity levels^5^.

Level	Severity definition	HA	SL	AL	CO	FE	HO	ST	CP	FA	CN	MP	SB	DI	AA
1	‘Flu-like’ with no fever	$\checkmark$	$\checkmark$	–	$\checkmark$	–	–	$\checkmark$	$\checkmark$	–	–	$\checkmark$	–	–	–
2	‘Flu-like’ with fever	$\checkmark$	$\checkmark$	$\checkmark$	$\checkmark$	$\checkmark$	–	$\checkmark$	–	$\checkmark$	–	–	–	–	–
3	Gastrointestinal	$\checkmark$	$\checkmark$	$\checkmark$	–	–	–	$\checkmark$	$\checkmark$	–	–	–	–	–	–
4	Severe level one; fatigue	$\checkmark$	$\checkmark$	–	$\checkmark$	$\checkmark$	–	$\checkmark$	$\checkmark$	$\checkmark$	–	–	–	–	–
5	Severe level two; confusion	$\checkmark$	$\checkmark$	$\checkmark$	$\checkmark$	$\checkmark$	$\checkmark$	$\checkmark$	-	$\checkmark$	$\checkmark$	$\checkmark$	–	–	–
6	Severe level three; Abdominal and respiratory	$\checkmark$	$\checkmark$	$\checkmark$	$\checkmark$	$\checkmark$	$\checkmark$	$\checkmark$	$\checkmark$	$\checkmark$	$\checkmark$	$\checkmark$	$\checkmark$	$\checkmark$	$\checkmark$

*HA* Headache, *SL* loss of smell, *AL* loss of appetite, *CO* cough, *FE* fever, *HO* hoarseness, *ST* sore throat, *CP* chest pain, *FA* fatigue, *CN* confusion, *MP* muscle pain, *SB* shortness of breath, *DI* diarrhea, *AA* abdominal pain.

Given the concerns above, we study the following two primary research questions (*RQ*):

- (RQ1): How can user-friendly interfaces and visualisation techniques be integrated into clinical decision support systems to facilitate user interaction and interpret COVID-19 symptom data?
- (RQ2): How can we model uncertain and complex inter-dependencies between Covid-19 symptoms?
- (RQ3): How can we validate the prediction of the severity of COVID-19 and its determinants probabilistically?

To our knowledge, no previous study has adequately addressed the multifaceted challenges of COVID-19 management through comprehensive clinical decision support systems. Existing research often lacks a focus on integrating user-friendly interfaces and visualization techniques to facilitate user interaction and interpretation of COVID-19 symptom data. Given all of these details, the following are the key contributions of this study to answering the research questions:

- To address *RQ1*, we develop intuitive user interfaces and visualisation tools within BayesCovid, which enable healthcare professionals to interact with and interpret COVID-19 symptom data effectively to enhance user engagement and facilitate informed decision-making in clinical settings.
- To address *RQ2*, we propose an entirely automated and data-driven system, BayesCovid, that integrates both traditional Bayesian network learning models-such as *Naïve Bayesian Network*, *Tree-Augmented Naïve Bayesian Network*, and *Complex Bayesian Network*-and Bayesian deep learning models. This unified approach automates all operations, including data preprocessing, and models uncertain and complex dependencies between COVID-19 symptoms. By deploying Bayesian deep learning models within BayesCovid, we leverage their capabilities to overcome traditional Bayesian network limitations by enhancing the learning of feature representations.
- To address *RQ3*, we validate our probabilistic computational method powered by predefined functions to predict the COVID-19 severity and its determinants. Our experimental results show that BayesCovid can be used to uncover the hidden and complex patterns of the COVID-19 pandemic with accuracy rates of between 83.52 and 87.35%.

## Background

### Coronavirus pandemic (COVID-19) in the world

**Figure 1:**
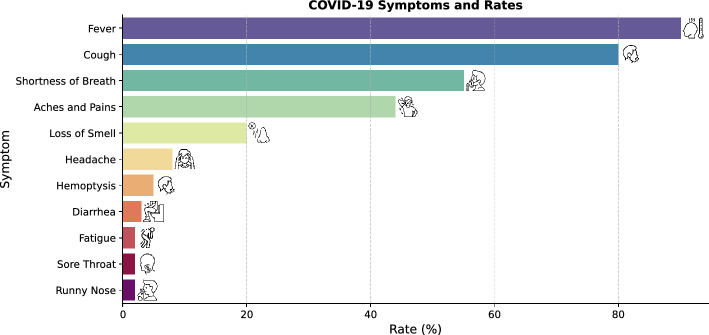
Percentage of people with COVID-19 symptoms (Source of Icons: https://www.pngwing.com/).

Person-to-person transmission of SARS-CoV2 became a global problem in 2020, with those infected with mild to moderate respiratory illnesses. Due to the alarming spread of the COVID-19 epidemic in the World, the World Health Organization (WHO) declared COVID-19 a pandemic in March 2020 and made standard recommendations, such as regular hand washing, covering the mouth and nose when coughing and sneezing, coughing and avoiding close contact with people who show signs of respiratory illness such as sneezing^6^. Figure 1 shows the most common symptoms of 55.924 laboratory-confirmed cases of COVID-19, reported by China in February 2020^7^. According to the report published on October 21, 2022^8^, approximately 632,055,653 confirmed cases, 610,853,327 recovered cases, and 6,579,814 deaths occurred worldwide until October 21, 2022.

### Bayesian network for knowledge discovery

Bayesian networks, called probabilistic networks, are well-known and adaptable models that simulate complicated interaction systems at the nexus of statistics and ML^9^. Using a network topology in which the measured characteristics are the nodes and the directed edges indicate the interactions between those nodes, BNs directly explain multivariate interdependencies^10^. Due to their interpretable structure, they differ from “black-box” notions used in other machine-learning techniques. Contrary to logistic regression, statistical dependency and independence in Bayesian networks are directly expressed rather than being obscured by approximate weights as they are in logistic regression.

A conditional probability is the likelihood of a conclusion, $\eta$, given a piece of information or an observation, $\gamma$, where $\eta$ and $\gamma$ are interdependent. The symbol for this probability is Pr($\eta$ | $\gamma$), where:

1 $$\begin{aligned} Pr(\gamma | \eta ) = \frac{(Pr(\eta | \gamma )).Pr(\gamma )}{Pr(\eta )} \end{aligned}$$

Bayes’ theorem is a technique for determining the conditional’s opposite probability. This conditional relationship reveals probability information about $\eta$ or $\gamma$ with the known outcome of the other as seen in Eq. (2):

2 $$\begin{aligned} Pr(\eta | \gamma ) = \frac{(Pr(\gamma | \eta )).Pr(\eta )}{Pr(\gamma )} = \frac{(Pr(\gamma ),\eta )}{Pr(\gamma )} \end{aligned}$$

The joint probability distribution is a fundamental concept underlying the inference process in Bayesian networks. The joint probability distribution encapsulates the simultaneous likelihood of all system variables, which reflects their interdependencies as modelled by the directed acyclic graph (DAG), quantifying the probability of observing a particular combination of states for all variables in the network. By leveraging the conditional independence relationships encoded in the Bayesian network structure, the joint probability distribution can be factorised into a product of conditional probabilities that facilitate efficient and coherent reasoning about the system.

### Bayesian network models

We implemented three well-known Bayesian network models, namely *Naïve Bayesian Network, Tree-Augmented Naïve Bayesian Network*, and *Complex Bayesian Network*. Moreover, we combine these algorithms with deep learning for further investigation. We implemented these algorithms in Python programming language (version 3.11.5) (https://www.python.org/downloads/release/python-3115/) using *pgmpy* (https://github.com/pgmpy/pgmpy) library, a Bayesian Networks implementation used to work with probabilistic graphic models.

#### Naïve Bayesian network

This algorithm is a Naïve structure learning technique exclusively categorised as a structure learning algorithm since it directly constructs a Bayesian network from data, including its structure and parameters.

#### Tree-augmented Naïve Bayesian network

Tree Augmented Naive Bayesian is yet another structure learning algorithm that is similar to Naive Bayes, where the class variable is the parent of all remaining features (nodes), but the others are also interconnected, which aims to uncover possible dependence between the feature variables. These interconnections between the features are built dependent on the class variable.

#### Complex Bayesian network

Unlike other algorithms, the class variable can be a parent and child variable With the Complex Bayesian network. The features can be affected by the class variable and also can affect it.

#### Bayesian deep learning: enhancing feature representations and uncertainty estimation

Representations and Uncertainty Estimation Bayesian Deep Learning (BDL) uniquely combines the benefits of deep neural networks and Bayesian inference as a paradigm-shifting approach. BDL outperforms traditional deep learning approaches that offer deterministic predictions by explicitly incorporating and quantifying uncertainty inside its predictions. This capacity comes in handy while making decisions because it helps to grasp the degree of uncertainty. Traditional Bayesian network algorithms, such as Naïve Bayes, Tree-Augmented Naïve Bayesian, and Complex Bayesian, also grapple with uncertainty but often exhibit limitations in modelling complex, high-dimensional data. In comparison, BDL surpasses traditional deep learning methods in uncertainty estimation and enhances feature representations. BDL offers a advantage in learning and representing intricate relationships within data by incorporating Bayesian principles at the core of neural network weight modelling.

### Advantages of Bayesian network

Bayesian networks provide an intuitive and systematic approach for integrating preexisting knowledge with observed data within a well-established decision theory framework, as highlighted in a previous study by Stephenson et al.^11^. Bayesian networks offer accurate beliefs conditional on the facts without relying on asymptotic approximation. Without utilising the “plug-in” approach, Bayesian analysis can also directly estimate any parameters’ functions. The probability principle is followed: If two different sample strategies provide proportionate likelihood functions for $\theta$, both approaches should yield the same conclusions regarding $\theta$^12^. The probability principle is generally ignored by classical reasoning. Furthermore, Bayesian networks offer a suitable environment for various models, including hierarchical models and issues with missing data.

## Proposed method: BayesCovid

**Figure 2:**
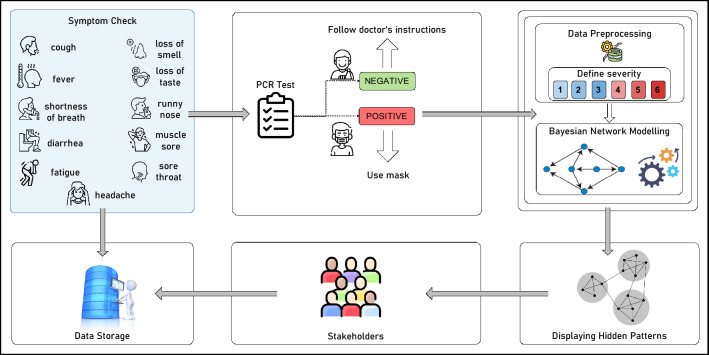
The high-level architecture of BayesCovid (Source of Icons: https://www.pngwing.com/.

The proposed system is depicted in Fig. 2. The collected dataset is sent to the preprocessing module to prepare it for implementing Bayesian network algorithms. The predefined functions define the severity of COVID-19 based on the criterias identified by Sudre et al.^5^. Afterwards, different Bayesian network algorithms, namely *Naïve Bayesian Network, Tree-Augmented Naïve Bayesian Network*, *Complex Bayesian Network* and Bayesian deep learning algorithms are applied to the dataset to uncover the interdependencies between the features with probabilities. Before sharing and storing the results, the hidden patterns are displayed.

### Distinctive features and integration capabilities of BayesCovid

BayesCovid distinguishes itself from existing Bayesian network structures and parameter learning packages through several unique features that make it a valuable resource for the clinical community. First and foremost, BayesCovid ’s seamless integration with big data systems, such as Apache Hadoop and Apache Spark, ensures its scalability to handle millions of patient records and petabytes of data, which makes it well-suited for large-scale epidemiological studies and hospital systems. Additionally, BayesCovid offers flexibility with the capability to run in local mode, allowing for diverse deployment environments. The system’s automated data preprocessing and COVID-19 severity identification streamline clinical workflows by reducing the need for manual intervention, thus saving valuable time for healthcare professionals. Moreover, the combination of Bayesian networks and deep learning techniques within BayesCovid enhances its predictive accuracy and ability to uncover complex symptom relationships, providing actionable insights that can significantly improve clinical decision-making and patient outcomes. To further facilitate adoption and usability, we share the source code of BayesCovid, well-explained documentation, and a demonstration video (see “Tool availability” section) to enable easy implementation and a comprehensive understanding of the tool’s functionalities and benefits. These features collectively position BayesCovid as an advanced, scalable, and practical tool for addressing the dynamic challenges of the COVID-19 pandemic in various clinical settings.

### Data preprocessing

#### Data integration

This study utilised a comprehensive dataset (https://github.com/mdcollab/covidclinicaldata) from the Coronavirus Disease 2019 (COVID-19) Clinical Data Repository, a valuable compilation of clinical characteristics from individuals who underwent COVID-19 testing. The dataset contains information such as epidemiologic factors, comorbidities, vitals, clinician-assessed, and patient-reported symptoms. Additionally, the dataset incorporates radiological and laboratory findings while ensuring compliance with the HIPAA Privacy Rule’s De-Identification Standard to safeguard patient privacy. The data is a joint effort of Carbon Health, which provides clinical characteristics and laboratory findings, and Braid Health, contributing chest X-rays, findings, labels, and clinician impressions. The dataset is provided in CSV format, consisting of 29 files. This step combines the data in different files with the same data structure into a single file by executing preprocessing.

#### Data imputation

This step is applied to manage missing values. After data integration/combination, we acquired a total of 93,995 COVID-19 data with the symptoms, each of which had a missing ratio of around 0.1%, except fever which the missing value is about 24%. To this end, we removed rows with missing values to ensure high accuracy and consistency. Finally, our dataset consists of 18,538 COVID-19 case data.

#### Encoding the categorical data

We implemented label encoding to digitalize the text-based information values (true/false) in the dataset, which converts the data into integer values (0/1).

#### Feature extraction

Using the information in Table 1, we obtain the COVID-19 severity for each case ranging from 1 to 6.

### Data discretization

Data discretization assigns a particular data value to each interval after converting attribute values from continuous data into a constrained collection of intervals^13^. We discretized the data to maximize the mutual information between parent and child nodes. Therefore, we specified the optimal discretization threshold for each variable in our dataset over two values, 0 and 1.

## Evaluation and results analysis

This section presents the experimental results and comprehensive evaluations of BayesCovid. We will explicitly discuss the results of the algorithms applied to the clinical datasets to uncover the hidden patterns of COVID-19 symptoms.

### Experimental setup

#### Environments

We set up a Spark on Hadoop Yarn cluster consisting of 4 EC2 machines, 1 master and 3 workers in AWS to deploy BayesCovid. We chose Ubuntu Server 20.04 LTS as the operating system for all the machines and installed Hadoop version 3.3.2 and Spark 3.3.1. All the nodes have 4 cores and 16 GB of memory.

#### Dataset

The dataset, prepared by Carbon Health and Braid Health^14^, was obtained through RT-PCR tests from 11,169 individuals, approximately 3% of patients living in the United States who had COVID-positive, and 97% had COVID-negative tests. This dataset, which began to be collected by Carbon Health in early April 2020, was collected under the anonymity standard of the Health Insurance Portability and Accountability Act (HIPAA) privacy rule. This dataset covers multiple physiognomies, including Epidemiological (Epi) Factors, comorbidity, vital signs, healthcare worker-identified, patient-reported, and COVID-19 symptoms. In addition, information about patients, such as heart rate, temperature, diabetes, cancer, asthma, smoking and age, is also available. The Carbon Health team gathered the Braid Health team datasets, which collected radiological information, including CXR information. This dataset includes data from patients with one or more symptoms and no symptoms, and we only used the **COVID-19** symptom information indicated in Fig. 1. Radiological information was not included in the analysis. Table 2 shows the statistical information of the COVID-19 dataset. We have 18,538 test results of 11 different COVID-19 symptoms and COVID severity values, belonging to 11,169 individuals. Moreover, Table 3 demonstrates the number of false (negative) and true (positive) values for each symptom.

**Table 2:** Statistics related to symptom values presented in the dataset.

Symptoms	Mean	Std. dev.	Min.	Max.	Count
Cough	0.121	0.326	0	1	18538
Fever	0.048	0.214	0	1	18538
Shortness of breath	0.058	0.235	0	1	18538
Diarrhea	0.033	0.181	0	1	18538
Fatigue	0.116	0.321	0	1	18538
Headache	0.100	0.301	0	1	18538
Loss of smell	0.011	0.107	0	1	18538
Loss of taste	0.013	0.115	0	1	18538
Runny nose	0.063	0.243	0	1	18538
Muscle sore	0.066	0.249	0	1	18538
Sore throat	0.117	0.321	0	1	18538
COVID severity	0.041	0.359	0	6	18538

**Table 3:** Data counts representation using hierarchical discretization for symptoms.

Value	Cough	Fever	Shortness of breath	Diarrhea	Fatigue	Headache	Loss of smell	Loss of taste	Runny nose	Muscle sore	Sore throat
False	16288	17641	17445	17908	16374	16666	18321	18289	17364	17303	16369
True	2250	897	1093	630	2164	1872	217	249	1174	1235	2169

### Cross validation

Cross-validation is an important step in assessing the predictive power of models while mitigating the risk of overfitting^15^. To rigorously evaluate our models, we implemented ten-fold cross-validation by dividing the dataset into ten equal parts. During each iteration, one part was the validation/test set, while the remaining nine were used for model training. This process was repeated ten times, and the resulting accuracies were averaged across all folds to assess each model’s performance comprehensively. Importantly, using ten-fold cross-validation ensures that every instance in the dataset is precisely used once as a testing and training sample, which minimises the risk of overfitting^16^.

### Implemented Bayesian network algorithms

This subsection explains three distinct Bayesian networks: Naïve Bayesian, Tree-Augmented Naïve Bayesian, and Complex Bayesian models. These models have unveiled intricate and concealed patterns within COVID-19, offering valuable insights into the complex dynamics and relationships underlying the disease.

**Figure 3:**
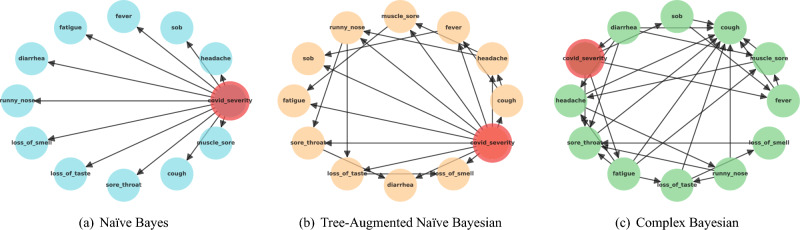
Dependency networks.

#### Naïve Bayesian algorithm

**Figure 4:**
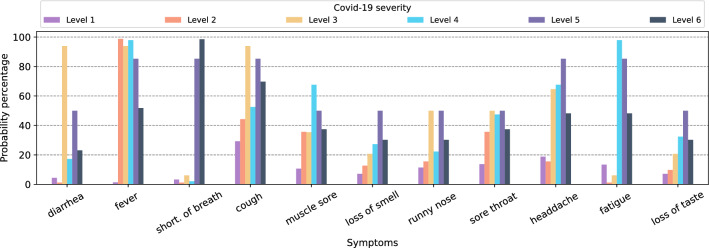
Conditional probability of symptoms with COVID-19 severity if symptoms are positive for Naïve Bayes.

**Figure 5:**
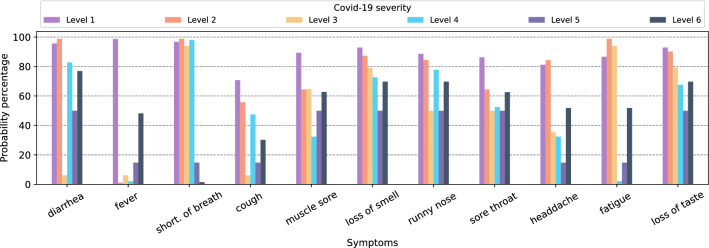
Conditional probability of symptoms with COVID-19 severity if symptoms are negative for Naïve Bayes.

Figure 3a depicts the dependencies for the Naïve Bayesian algorithm where the class variable, *COVID severity*, is the only parent associated with each symptom, and there is no link between symptoms. Figure 4 and 5 show the probability percentages of the symptoms for their positive and negative values. For example, in Fig. 4, while the probability of diarrhea is around 3% for COVID severity level 1, the probability of this symptom for level 3 is about 95%. Moreover, the probabilities of shortness of breath for levels 1, 2, 3, and 4 are very low, about 5%, and the likelihood of having this symptom is very high for levels 5 and 6. In short, the distribution of symptoms differs according to the severity levels of COVID-19, and the probability of some increases as the COVID-19 severity level rises. When we compare Figs. 4 and 5, it is seen that there is an inverse relationship between the incidence and absence of symptoms.

#### Tree-augmented Naïve Bayesian algorithm

The dependency network built using the Tree-augmented Naïve Bayesian network is depicted in Fig. 3b. COVID severity is the class variable similar to Naïve Bayesian network, but the connections between the symptoms (features) are also available. As seen from the figure, for example, *cough* has an effect on both *headache* and *fever* while *muscle sore* is affected by *headache* and affects *fatigue*. For the probabilities, Tables 4 and 5 show some results of the Conditional Probability Table (CPT). In Table 4, when *shortness of breath* and *fever* are negative (F), the probability of COVID severity level 1 is 92.95%. In contrast, when *shortness of breath* is positive (T), and *fever* is negative, then the probability of COVID severity level 1 is 2.04%. When *headache* is positive, but *cough* is negative, then the probability of COVID severity level 4 is 65.92% (see Table 5).

**Table 4:** CPT between three different symptoms and COVID severity using Tree-augmented Naïve Bayesian algorithm.

c_sev	c_sev (1)	c_sev (1)	c_sev (2)	c_sev (2)	c_sev (3)	c_sev (3)	c_sev (4)	c_sev (4)	c_sev (5)	c_sev (5)	c_sev (6)	c_sev (6)
Fever	Fever (F)	Fever (T)	Fever (F)	Fever (T)	Fever (F)	Fever (T)	Fever (F)	Fever (T)	Fever (F)	Fever (T)	Fever (F)	Fever (T)
sob (F)	92.95%	6.09%	50.0%	99.39%	50.0%	96.75%	50.0%	98.92%	50.0%	8.62%	1.55%	1.44%
sob (T)	2.04%	93.90%	50.0%	0.60%	50.0%	3.24%	50.0%	1.07%	50.0%	91.37%	98.44%	98.55%

*c_sev* COVID severity, *sob* shortness of breath, *F* false, *T* true.

**Table 5:** CPT between three different symptoms and COVID severity using Tree-augmented Naïve Bayesian algorithm.

Cough	Cough (F)	Cough (F)	Cough (F)	Cough (F)	Cough (F)	Cough (F)	Cough (T)	Cough (T)	Cough (T)	Cough (T)	Cough (T)	Cough (T)
c_sev	c_sev (1)	c_sev (2)	c_sev (3)	c_sev (4)	c_sev (5)	c_sev (6)	c_sev (1)	c_sev (2)	c_sev (3)	c_sev (4)	c_sev (5)	c_sev (6)
h_ache (F)	91.14%	93.77%	50.0%	34.07%	50.0%	50.0%	57.29%	72.70%	34.41%	30.80%	8.62%	52.57%
h_ache (T)	8.88%	6.22%	50.0%	65.92%	50.0%	50.0%	42.70%	27.29%	65.58%	69.2%	91.37%	47.42%

*c_sev* COVID severity, *sob* shortness of breath, *F* false, *T* true.

#### Complex Bayesian algorithm

Figure 3c shows the dependencies between all the symptoms (features) and COVID severity (class variable). Cough is most affected by different symptoms and does not affect any features. While the class variable, COVID severity, has impacts on *shortness of breath*, *fever*, *fatigue*, and *sore throat*, interestingly, it is affected by *diarrhea*. Another interesting pattern different from the Tree-augmented Naïve Bayesian network is that *fever* affects *muscle sore*. While Table 6 shows the CPT for three variables, namely *COVID severity*, *shortness of breath*, and *fever*, Table 7 displays the probabilities for four variables, namely *diarrhea*, *fatigue*, *muscle sore*, and *headache*. When *shortness of breath* is negative, and *fever* is positive, the probability of COVID severity level 4 is 98.92%. In contrast, when *shortness of breath* is positive, but *fever* is negative, the probability of COVID severity level 6 is 48.18% (see Table 6). For the probabilities based on the situation of four symptoms (see Table 6), for instance, when all three symptoms, *diarrhea*, *fatigue*, and *muscle sore*, are positive, the probability of having headache symptom is 73.18%. Another remarkable finding in Table 7 is that if an individual has *fatigue*, *muscle sore*, and *headache*, the probability of not having *diarrhea* is 58.43%.

**Table 6:** CPT between three different symptoms and COVID severity using Complex Bayesian algorithm.

c_sev	c_sev (1)	c_sev (1)	c_sev (2)	c_sev (2)	c_sev (3)	c_sev (3)	c_sev (4)	c_sev (4)	c_sev (5)	c_sev (5)	c_sev (6)	c_sev (6)
sob	sob (F)	sob (T)	sob (F)	sob (T)	sob (F)	sob (T)	sob (F)	sob (T)	sob (F)	sob (T)	sob (F)	sob (T)
Fever (F)	99.91%	61.88%	0.60%	50.0%	3.24%	50.0%	1.07%	50.0%	50.0%	8.62%	50.0%	48.17%
Fever (T)	0.08%	38.11%	99.39%	50.0%	96.75%	50.0%	98.92%	50.0%	50.0%	91.37%	50.0%	51.82%

*c_sev* COVID severity, *sob* shortness of breath, *F* false, *T* true.

**Table 7:** CPT between four different symptoms and COVID severity using Complex Bayesian algorithm.

Diarrhea	Diarrhea (F)	Diarrhea (F)	Diarrhea (F)	Diarrhea (F)	Diarrhea (T)	Diarrhea (T)	Diarrhea (T)	Diarrhea (T)
Fatigue	Fatigue (F)	Fatigue (F)	Fatigue (T)	Fatigue (T)	Fatigue (F)	Fatigue (F)	Fatigue (T)	Fatigue (T)
m_sore	m_sore (F)	m_sore (T)	m_sore (F)	m_sore (T)	m_sore (F)	m_sore (T)	m_sore (F)	m_sore (T)
h_ache (F)	89.43%	52.11%	48.19%	41.56%	50.0%	23.33%	28.37%	26.81%
h_ache (T)	10.56%	47.88%	51.80%	58.43%	50.0%	76.66%	71.62%	73.18%

*m_sore* muscle sore, *h_ache* headache, *F* false, *T* true.

#### Implemented Bayesian deep learning algorithms

**Figure 6:**
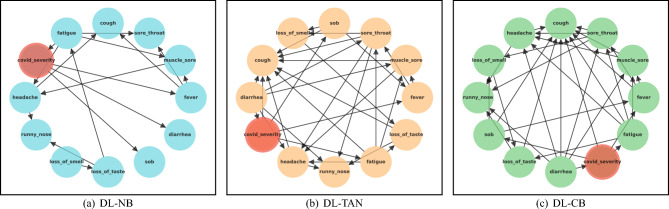
Bayesian deep learning dependency networks.

In this study, we have also investigated and implemented three distinct Bayesian models, each representing a unique intersection of deep learning and Bayesian inference. The first model, Deep Learning-based Naïve Bayes (DL-NB), is a deep learning-based Naïve Bayes structure that capitalises on the capacity of deep neural networks to refine the traditional Naïve Bayes model for enhanced feature learning and dependency representation. Additionally, we extended our exploration to traditional Bayesian network structures by implementing Deep Learning-based Tree-Augmented Naïve Bayes (DL-TAN), where deep learning principles are integrated to augment the classic Tree-Augmented Naïve Bayes algorithm, providing richer feature representations. Furthermore, our investigation includes Deep Learning-based Complex Bayesian (DL-CB), a model designed to overcome the limitations of traditional Complex Bayesian structures in modelling intricate relationships within high-dimensional data. This comprehensive analysis and implementation of DL-NB, DL-TAN, and DL-CB contribute to the broader understanding of the synergies between deep learning and Bayesian techniques in various Bayesian network architectures. Figure 6 demonstrates the network dependencies of deep learning-based Bayesian network algorithms which uncover the complex and hidden relationships between COVID symptoms. As illustrated in Fig. 6a–c, our Bayesian deep learning models, namely DL-NB, DL-TAN, and DL-CB, reveal a richer web of relationships among features compared to their traditional counterparts. The Bayesian Deep Learning models exhibit a higher density of connections, which indicates a more nuanced understanding of inter-feature dependencies. This heightened connectivity means the enhanced capacity of Bayesian Deep Learning to capture complex relationships within the data that provide a comprehensive and informative modelling of the underlying dynamics.

#### Evaluation of the accuracy of Bayesian network algorithms

**Figure 7:**
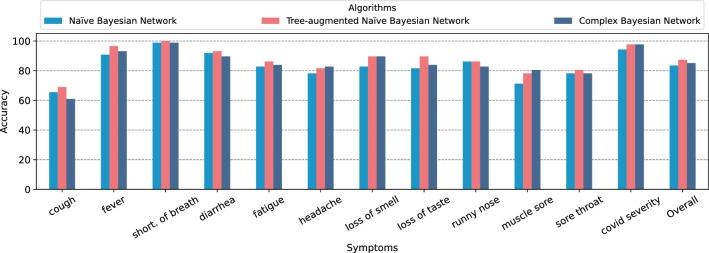
Total accuracy’s of the algorithms.

Figure 7 demonstrates the accuracy’s for the three different algorithms proposed in our system, namely *Naïve Bayesian Network, Tree-Augmented Naïve Bayesian Network*, and *Complex Bayesian Network*. Although the general accuracy of the algorithms is close to each other, there are apparent differences in the accuracy of the symptoms. The algorithms perform between 60% and 68% poorly for cough symptoms, while they show high accuracy’s for COVID severity ranging from 94% to 97%. The overall accuracy’s of these three algorithms are 83.52%, 87.35%, and 85.15%, respectively.

#### Evaluation of the accuracy of Deep learning-based Bayesian network algorithms

**Figure 8:**
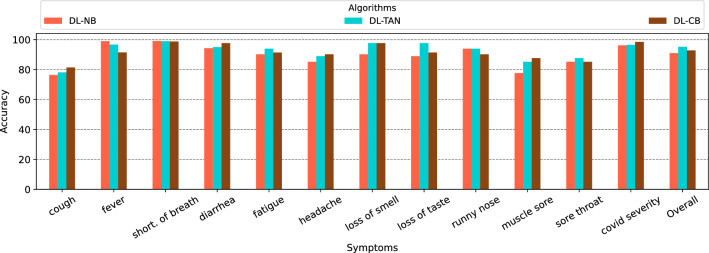
Total accuracy’s of the deep learning-based Bayesian algorithms.

In the evaluation of the accuracy of deep learning-based Bayesian network algorithms, the results, as depicted in Fig. 8, showcase the performance of three distinct models: DL-NB, DL-TAN, and DL-CB. The overall accuracies reveal nuanced differences among the algorithms. DL-TAN emerges with the highest cumulative accuracy of 95.21%, which indicates its superior predictive capabilities across a spectrum of symptoms. DL-NB and DL-CB follow closely, exhibiting overall accuracies of 91.04% and 92.81%, respectively. These results underscore the efficacy of deep learning-based Bayesian approaches in capturing complex relationships within the dataset.

The comparative analysis of Bayesian deep learning algorithms against traditional Bayesian network algorithms elucidates a discernible advantage favouring the former. Notably, the Bayesian deep learning models, such as DL-NB, DL-TAN, and DL-CB, exhibit superior predictive performance across various symptoms.

### Web interface

We have developed a web interface for BayesCovid decision support system that can be used by any clinical practitioner or other users. It utilises Python libraries, concerning probabilistic graphical models, data manipulation, network analysis, and data visualization. Additionally, tkinter is adopted for the graphical user interface, and PyMuPDF (fitz) is leveraged for PDF file handling. All the source code and accompanying documentation for BayesCovid decision support system are available as open-source on GitHub (https://github.com/umitdemirbaga/BayesCovid). A demonstration is also available online on YouTube (https://youtu.be/7j36HuC9Zto). The designed user interface provides dual functionality highlighted below.

**Figure 9:**
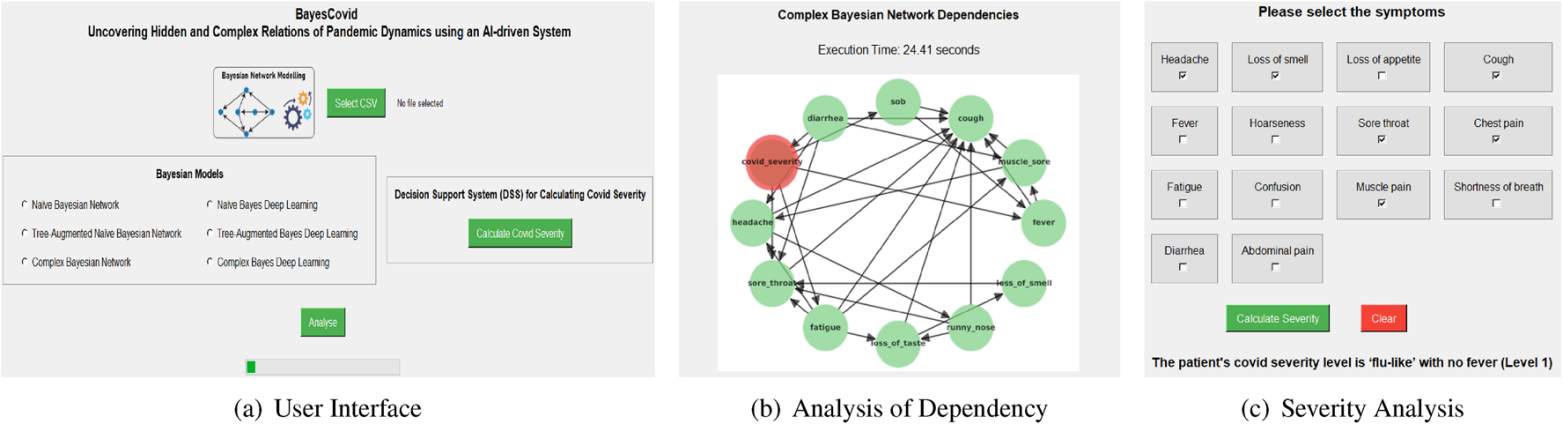
User interface visuals.

- Dependency analysis: This component of application ensures efficient and accurate relationship analysis between the symptoms and severity assessment, enhancing the decision-making process in clinical settings. Figure 9a depicts the user-friendly interface where a data file can be uploaded using “Select CSV” button. After the data file is uploaded, six radio buttons are provided for users to select one of the following Bayesian models: (a) Naïve Bayesian Network, (b) Tree-Augmented Naïve Bayesian Network, (c) Complex Bayesian Network, (d) Naïve Bayes Deep Learning, (e) Tree-Augmented Bayes Deep Learning, and (e) Complex Bayes Deep Learning. An “Analyse” button that starts the processing of the selected model with the selected CSV file. A progress bar populates to show the processing status. After the model is processed, the dependency network plot is generated (see Fig. 9b) and the CPT output is saved as a file.
- Severity analysis: This component of the application assists clinical staff in calculating the severity of COVID-19. This feature assists in selecting the detected symptoms that the patient exhibits and subsequently determines the severity of COVID-19. As depicted in Fig. 9c a clinician or user can select the visible symptoms and calculate severity. This will output the COVID-19 severity level based on the input symptoms as shown Fig. 9c.

## Related work

### Predictive models for COVID-19 diagnosis

With the emergence of the COVID-19 pandemic, predictive models have commonly been applied to ease the burden on health systems and to enable more accurate analysis. These models have been widely used to diagnose COVID-19 disease and predict injection risk and spread. For example, Batista et al.^17^ implement different ML algorithms (neural networks, random forests, gradient boosting trees, logistic regression, and SVM) over emergency care admission data from 235 adult patients to predict the risk of positive COVID-19 diagnosis. Similarly, the authors of^18–20^ use AI techniques in chest imaging data to predict COVID-19 infection. ML techniques have been implemented to discover the risk variables linked to the death of coronavirus-infected people in^21,22^. Jiang et al.^23^ propose an AI-based framework with predictive analytics that analyzes clinical risk factors to predict mortality due to COVID-19. Wu et al.^24^ propose an ML-based critical risk assessment system to predict the COVID-19 severity risk for patients at hospital admission. The authors of^25^ use Multivariate Cox regression to elucidate high-risk indicators for coronavirus disease 2019 (COVID-19) and create a prediction model of illness development to assist doctors in making more informed treatment decisions. Soltan et al. develop a model for the triage of COVID-19 patients awaiting RT-PCR screening by applying ML models such as random forest, logistic regression, and hyper-sloped trees to data from hospitalized patients with severe COVID-19 symptoms. Menni et al.^26^ propose a framework using logistic regression statistical model to predict COVID-19 infection based on potential COVID-19 symptoms, such as anosmia, diarrhea, shortness of breath, cough, skipped meals, hoarse voice, abdominal pain, fever, chest pain, diarrhea, delirium. Unfortunately, this data, collected with a mobile application, is insufficient to discover symptoms based on empirical evidence, as it only works based on these symptoms. However, these predictive models do not consider modelling the relationship between COVID-19 stochastic symptoms in a probabilistic manner, most of which have concentrated on prognostic factors for survival.

### Bayesian networks for knowledge discovery

Leveraging Bayesian probabilistic reasoning, Bayesian networks support clinical decision-making on patients’ health status, considering uncertainties^27^. Gaglione et al.^28^ develop an estimation and forecasting model based on the Bayesian decision theory approach, called Bayesian sequential estimation, to estimate the current situation and underlying causes of the COVID-19 epidemic along with the evolution of the epidemiological curve. In this study^29^, the authors predict the evolution of the COVID-19 pandemic in Spain by combining deep learning techniques with the Bayesian Poisson-Gamma model. While DL describes SARS-CoV-2 sequencing, the Poisson-Gamma model reveals the posterior predictive distribution of counts. Thus, the variation of sequences in the entire region is predicted and predicts the outcomes of possible scenarios. Saqib^30^ estimates the COVID-19 dependent variable using a mathematical model that combines the Bayesian Ridge Regression model with an n-order Polynomial. The author estimates the possible causes for the upcoming days, using data from different countries to validate the model. The authors in^31^ propose a system that integrates Bayesian networks with supervised ML algorithms to reveal what factors affect mental health during the COVID-19 pandemic. This study demonstrated the effects of symptoms, comorbidities, and changing economic factors on mental health. In addition, they estimate people’s susceptibility to anxiety attacks under these effects. Shen et al.^32^ propose a decision-making and risk assessment system that determines the risk of COVID-19 patients spreading the virus using Bayesian networks to assist physicians in decision-making and risk assessment during the Covid-19 pandemic. They used parameter learning and structure learning to design the bayesian networks model and tested the viability of this model using actual sample data. They compared the model with Random Forest, k-Nearest Neighbor, and Support Vector Machine algorithms based on accuracy, sensitivity, specificity and F − 1 score indicators. Using Bayesian networks, the authors in^33^ propose a Clinical Decision Support System for diagnosing respiratory diseases, including those caused by COVID-19. They created this system by using the prevalence of diseases, symptoms and laboratory results and tested it with the help of specialist physicians by exposing them to real clinical consultation cases.

## Conclusion and future work

Bayesian networks offer a probability distribution to reveal the relationship between variables and the impact of variables on the final result. In this work, we proposed a novel data-driven and automated decision support system, BayesCovid, built on Bayesian networks and deep learning to determine COVID-19 severity while identifying complex and hidden relationships between COVID-19 symptoms based on test results. Our approach combines a set of user-defined functions and different Bayesian network algorithms, and deep learning methods. Thus, data preprocessing and COVID-19 severity identification are performed automatically while the complexity of model interpretation is reduced. Our system, developed and tested with real-world clinical data, demonstrated high performance with accuracy ranging from 83.52 to 98.97%.

In future work, we plan to deepen our collaboration with domain experts that will involve implementing BayesCovid. This allows us to gain valuable insights into our system’s performance and practical utility in different clinical settings. In this way, we aim to conduct a qualitative evaluation by working closely with these experts to ensure BayesCovid aligns with real-world clinical needs and provides meaningful contributions to COVID-19 severity prediction. Our other aim is to expand the scope of BayesCovid by implementing and evaluating the system in different populations and regions, which will contribute to a more comprehensive understanding of the model’s performance across diverse demographic and geographic contexts.

## Data Availability

The datasets used/analysed during the current study are publically available in the ‘Coronavirus Disease 2019 (COVID-19) Clinical Data Repository’ at (https://github.com/mdcollab/covidclinicaldata). No data was generated in this research. All the source code and accompanying documentation for this tool are available as open-source on GitHub (https://github.com/umitdemirbaga/BayesCovid). A demonstration is also available online on YouTube (https://youtu.be/7j36HuC9Zto).
